# Impact of diabetes mellitus severity, treatment regimen and glycaemic control on atrial fibrillation prevalence in the Polish population aged ≥ 65

**DOI:** 10.1038/s41598-023-43939-5

**Published:** 2023-10-12

**Authors:** Jakub Gumprecht, Gregory Y. H. Lip, Adam Sokal, Beata Średniawa, Jakub Stokwiszewski, Tomasz Zdrojewski, Marcin Rutkowski, Tomasz Grodzicki, Jarosław Kaźmierczak, Grzegorz Opolski, Zbigniew Kalarus

**Affiliations:** 1 Liverpool Centre for Cardiovascular Science, University of Liverpool, Liverpool John Moores University and Liverpool Heart & Chest Hospital, Liverpool, UK; 2 Department of Clinical Medicine, Aalborg University, Aalborg, Denmark; 3 Department of Cardiology, DMS in Zabrze, Medical University of Silesia, Katowice, Poland; 4 Department of Cardiology, Silesian Centre for Heart Diseases, Zabrze, Poland; 5 Silesian Park of Medical Technology Kardio-Med Silesia in Zabrze, Zabrze, Poland; 6 National Institute of Hygiene, Warsaw, Poland; 7 Department of Preventive Medicine and Education, Medical University of Gdańsk, Gdańsk, Poland; 8 Department of Internal Medicine and Gerontology, Jagiellonian University Medical College, Kraków, Poland; 9 Department of Cardiology, Pomeranian Medical University, Szczecin, Poland; 10 First Chair and Department of Cardiology, Medical University of Warsaw, Warsaw, Poland

**Keywords:** Cardiology, Medical research, Risk factors

## Abstract

Diabetes mellitus (DM) is a well-known risk factor for atrial fibrillation (AF), but the mechanism(s) by which DM affects AF prevalence remains unclear. This study aims to evaluate the impact of diabetes mellitus severity (expressed as its known duration), antihyperglycemic treatment regimen and glycaemic control on AF prevalence. From the representative sample of 3014 participants (mean age 77.5, 49.1% female) from the cross-sectional NOMED-AF study, 881 participants (mean age 77.6 ± 0.25, 46.4% female) with concomitant DM were involved in the analysis. AF was screened using a telemonitoring vest for a mean of 21.9 ± 9.1 days. The mean DM duration was 12 ± 0.35 years, but no significant impact of DM timespan on AF prevalence was observed. No differences in the treatment pattern (oral medication vs insulin vs both oral + insulin) among the study population with and without AF were shown (p = 0.106). Metabolic control reflected by HbA1c levels showed no significant association with AF and silent AF prevalence (p = 0.635; p = 0.094). On multivariate analyses, age (Odds Ratio (OR) 1.35, 95%CI: 1.18–1.53, p < 0.001), p = 0.042), body mass index (BMI; OR 1.043, 95%CI: 1.01–1.08, p = 0.027) and LDL < 100 mg/dl (OR 0.64, 95%CI: 0.42–0.97, p = 0.037) were independent risk factors for AF prevalence, while age (OR 1.45, 95%CI: 1.20–1.75, p < 0.001), LDL < 100 mg/dl (OR 0.43, 95%CI 0.23–0.82, p = 0.011), use of statins (OR 0.51, 95%CI: 0.28–0.94, p = 0.031) and HbA1c ≤ 6.5 (OR 0.46, 95%CI: 0.25–0.85, p = 0.013) were associated with silent AF prevalence. Diabetes duration, diabetic treatment pattern or metabolic control per se did not significantly impact the prevalence of AF, including silent AF detected by prospective continuous monitoring. Independent predictors of AF were age, BMI and low LDL levels, with statins and HbA1c ≤ 6.5 being additional independent predictors for silent AF.

Trial registration: NCT03243474.

## Introduction

Diabetes mellitus (DM), the most prevalent metabolic disorder worldwide, is a well-known independent risk factor for cardiovascular adverse events and mortality^1^. The risk has not only been attributed to atherosclerosis or micro-/macro-vascular complications but also increased thromboembolic events associated with a higher prevalence of atrial fibrillation (AF) among the DM population^2–4^. The pathophysiological background underlying the frequent coexistence of diabetes and AF seems to be multifactorial, involving changes in left atrial size, abnormal proinflammatory mediators and endothelial dysfunction^5^.

Concomitant DM and AF constitute a substantial health burden leading to or worsening several conditions, including hypertension, stroke or heart failure^6–8^. Both disorders also interfere with haemostasis by favouring the prothrombotic effect of platelet aggregation, endothelial dysfunction, and impairment in coagulation and fibrinolysis processes^9–11^.

Although several studies have already been published in relation to the association between diabetes and prevalent AF, the reports seem to be ambiguous. Several studies suggest an increased AF prevalence in patients with recurrent hyperglycaemia^8,12^ and a beneficial effect of tight glycaemic control on the arrhythmia incidence^13^. Conversely, other reports show no influence of aggressive antidiabetic management on AF incidence^14–16^ or the increased risk was mainly assigned to a prolonged duration of diabetes^17^.

Accordingly, this study aims to evaluate the impact of diabetes mellitus severity (expressed as its known duration), antihyperglycemic treatment regimen, and glycaemic control on AF prevalence in the Polish population aged ≥ 65 years.

## Methods

The study was conducted as a sub-analysis of the Non-invasive Monitoring for Early Detection of Atrial Fibrillation (NOMED-AF) study, a cross-sectional observational study aiming to evaluate the AF prevalence and associated comorbidities in the Polish population. The detailed study protocol and methods has been previously described^18^. The study used a long-term wearable, non-invasive ECG monitoring system connected with an online data analysis and storage platform^18^.

The enrolment period was between Mar 15, 2017, and Mar 10, 2018. The trial schedule comprised multistage, stratified, and clustered population sampling, during which the representative Polish population aged ≥ 65 was stratified by province and place of residence. The procedure is thoroughly described in the supplementary appendix. Briefly, the whole country territory was stratified into 59 geographical strata. After that, the regions from each stratum (villages, towns, cities) were randomly selected by proportional probability. The study participants from the previously chosen areas were randomly chosen based on their identity number. Participants were divided into 5-year age groups. A similar number of men and women in each 5-year age group were designated. Therefore, we achieved the oversampling of older age groups. We did the process to ensure that the size of the final subsample of the most aged subjects would be enough for separate analyses. The oversampling was corrected at the stage of statistical analysis with weights to get population estimates. For each of the 3000 participants, another nine subjects living in the same cluster were drawn. These additional addresses were used in a predefined random order only if the address of the primarily chosen subject was incorrect or a subject refused to participate in the study^18^.

### Data collection

Each of the study participants was interviewed at home by a trained nurse using a standardized questionnaire. Relevant questions for the current analysis are as follows: previously diagnosed AF, symptoms and signs related to AF, symptoms of other cardiovascular diseases, and presence of concomitant diabetes mellitus or chronic kidney disease. There were also data collected to calculate the CHA_2_DS_2_-VASc score. Height and weight were taken from each participant. Blood pressure was measured during two separate visits at home using validated automated oscillometric devices. Urine and fasting blood samples were collected and processed in the central laboratory^18^. Due to the multicentre character of the study in each participating cardiovascular centre were a couple of trained nurses, which rotated randomly throughout the study to reduce the possible bias.

### Long-term ECG monitoring

Thirty-day, surface, 2-lead ECG recording was attempted in each of the study subjects, including respondents with already established AF diagnoses. Comarch Healthcare (Krakow, Poland) developed, manufactured and validated the dedicated ECG monitoring system specifically for the current study. The telemonitoring vest was validated in the first phase of the NOMED-AF study. During development, an internal validation was made according to the International Electrotechnical Commission standard 60601‐2‐47. The test was made on the MIT‐BIH Atrial Fibrillation Database that was not included in the algorithm development process to prevent overfitting. The database contains 25 records, each 10 h long. Thus, 93 h of AF episodes account for approximately 40% of the registration time. According to the standard, the episode sensitivity was 93%, and positive predictivity was 85%^19^. The system consisted of a vest equipped with ECG leads, two exchangeable recorders, and a docking station allowing to charge writers and transmit data. Another recorder was at the same time connected to the vest and recorded ECG data were sent to a central database with the use of GSM technology^18^.

The ECG recording was screened automatically for AF and atrial flutter episodes lasting longer than 30 s, using software developed and validated, especially for the purpose of the study. Episodes of atrial fibrillation/atrial flutter lasting longer than 30 s were automatically detected by AF detection algorithms of the analytical platform. Finally, each of the automatically detected episodes was reviewed by trained cardiologists^18^.

### Outcomes

The presence of AF was established based on the patient's medical records assessed for all subjects by the qualified study nurse on-site, confirmed by ECG record/monitoring (all participants had long-term ECG monitoring). AF de novo—newly diagnosed AF cases (not previously detected) were established based on up to 30 days of surface ECG monitoring for episodes of AF lasting 30 s or longer. Newly diagnosed AF (AF de novo) was defined as AF found in patients without previous history of this arrhythmia in available medical documentation. In this study, the term AF refers to both atrial fibrillation and/or atrial flutter^18^.

Patients were diagnosed with paroxysmal AF if the duration of the recorded longest arrhythmia event was shorter than 7 days and persistent/permanent AF if the arrhythmia duration exceeded 7 days. Because it is not always possible to distinguish precisely between persistent and permanent AF using patients' medical documentation, we analysed both as one group. Symptomatic AF was classified as arrhythmia in EHRA II-IV score and SAF as EHRA I^18^.

DM type 2 diagnosis was established in line with guidelines from the American Diabetes Association^20^ and European Association for the Study of Diabetes^21^ if the haemoglobin A1c (HbA1c) measured by HPLC was ≥ 6.5% or if the patient was aware of diabetes and a glucose-lowering treatment was applied or based on patients medical records. Physical activity threshold was defined as exercise at least > 30 min ≥ 3 times a week^22^.

None of the study participants were reported with DM type 1; hence, the analyses comprised only individuals diagnosed with type 2 diabetes. For the purpose of the current study, we distinguished three treatment regimens of diabetes: individuals on oral treatment only, those on oral treatment and insulin injections, and participants on insulin only.

Signed, informed consent was obtained from each eligible participant of the trial in accordance with protocol regulations approved by the local review boards governing research involving human subjects and the local bioethical committee in Katowice, Poland (number 26/2015), and the Declaration of Helsinki. The study was registered on clinicaltrials.gov (NCT03243474)^18^.

### Statistical analysis

Continuous variables were presented as mean and standard deviation (SD). Categorical variables were depicted as counts and percentages, analysed by chi-squared test. National estimation, i.e., the frequency of comorbidities prevalence, average values for age, BMI etc., were analysed on weighted data. The estimates were calculated so that the sample proportions were stratified by sex, age and city class were the same as in the Polish population. 95% Confidence intervals were determined, including the complex sampling scheme, and used to express the significance of differences between specific categories. Fisher's exact test was performed to compare differences between individual age categories. A logistic regression analysis was conducted to obtain the risk changes relative to age and sex. A multiple logistic regression analysis was performed to obtain independent risk factors of AF and SAF in DM+ and DM− populations. The independent variable was 5-year age groups and gender. A two-sided p-value < 0.05 was considered to be statistically significant.

Oversampling of elderly age groups was addressed using weights, which corrected the age and sex structure of the sample to the structure of the Polish population. Statistical analyses accounted for complex survey design. Prevalence and 95% confidence intervals (95%CI) were reported.

Finally, for each patient with paroxysmal AF, the number of hours of ECG monitoring before the first recorded AF event (lasting at least 30 s) was assessed. Based on these data, the relationship between the duration of ECG monitoring and the number of AF cases was examined.

### Ethics approval

Signed, informed consent was obtained from each eligible participant of the trial in accordance with protocol regulations approved by the local review boards governing research involving human subjects and local bioethical committee (26/2015), and the Declaration of Helsinki. The trial was registered on clinicaltrials.gov (NCT03243474).

## Results

Of the total 3014 participants (mean age 77.5, 49.1% female) from the NOMED-AF study, 881 subjects (29.2%; mean age 77.6 ± 0.25, 46.4% female) with concomitant diabetes mellitus were eligible and included in the analysis. Detailed baseline data are shown in Table 1.

**Table 1:** Baseline characteristics.

Clinical characteristics	DM +
	NOMED-AF population
	n [%]
65–69 years	142 [15]
70–74 years	197 [21]
75–79 years	189 [20]
80–84 years	168 [18]
85–89 years	118 [12]
≥ 90 years	67 [8]
Female	409 [46]
BMI (kg/m^2^)	29.77 ± 4.95
Hypertension	810 [92]
Heart Failure	242 [27]
Chronic kidney disease (eGFR < 60 ml/min)	285 [35]
Chronic kidney disease (TOTAL)	364 [40]
Haemodialysis	5 [5]
Stroke	118 [12]
Ischemic cerebral stroke	82 [9]
Intracranial haemorrhage	4 [0.5]
Unclassified stroke	32 [4]
TIA	65 [7]
Coronary heart disease	274 [29]
Myocardial Infarction	193 [21]
Peripheral artery disease	147 [16]
AF−	628 [71]
AF+	253 [28]
Silent AF	98 [13.5]
Paroxysmal	124 [13]
Persistent or permanent	129 [14]
CHA_2_DS_2_VASc (points)	5.08 ± 1.40
CHA_2_DS_2_VASc in AF patients	5.49 ± 1.37
CHA_2_DS_2_VASc in SAF patients	5.56 ± 1.45
DM duration (years)	12 ± 0.35
DM treatment regimen—oral treatment only	509 [73.7]
DM treatment regimen—oral treatment + insulin	79 [11.4]
DM treatment regimen—insulin only	103 [14.9]
HbA1c	6.90 ± 0.04

The mean ECG monitoring interval among the studied population was 21.9 ± 9.1 days, with the mean monitoring timespan among the DM + group was 20.31 ± 8.97 days and in the non-DM group, 19.97 ± 9.01 days. The mean time to detect any first AF episode was 7.25 ± 7.79 days, and for the first episode of silent AF, 8.48 ± 8.29 days.

### DM duration

**Figure 1:**
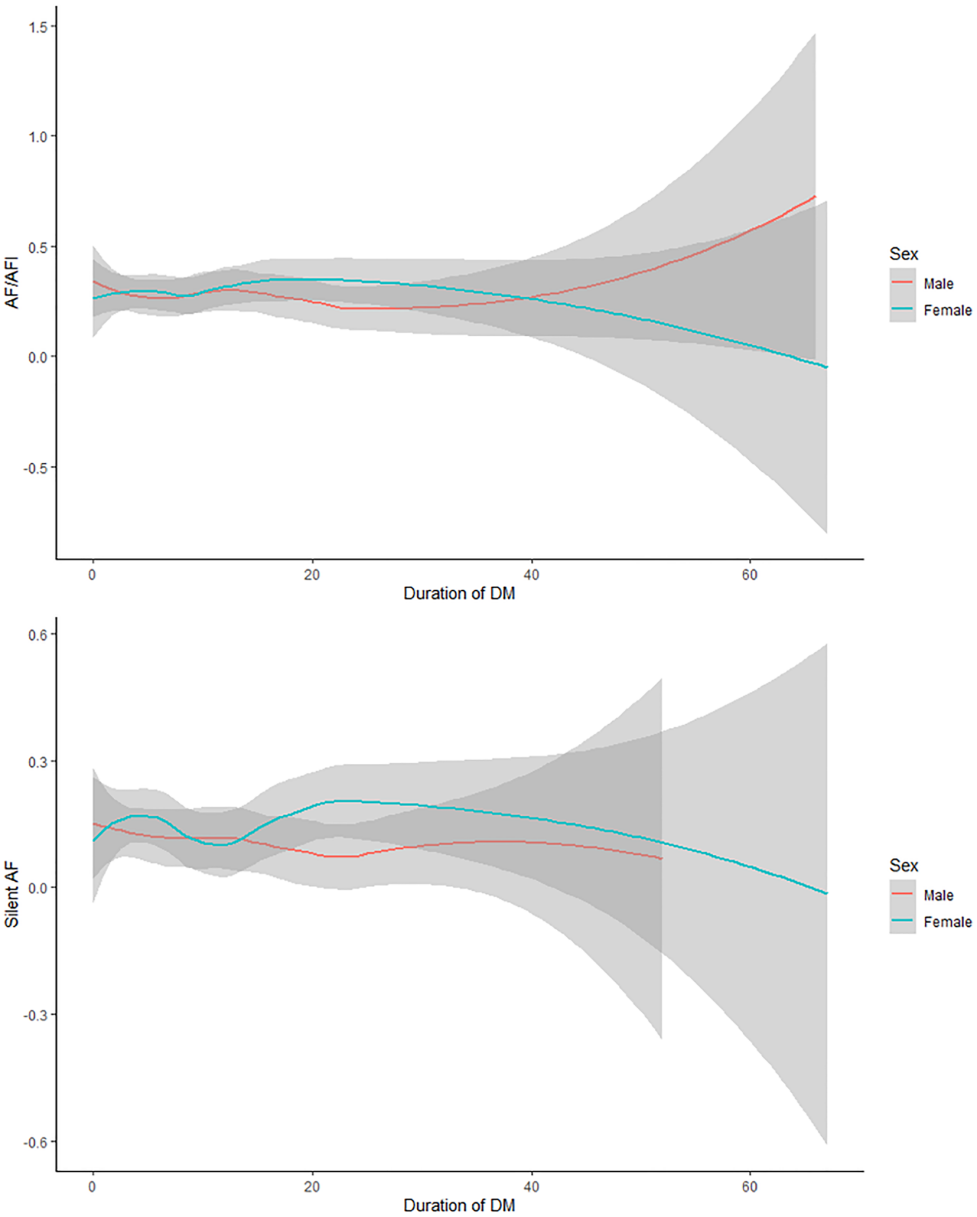
Atrial fibrillation and silent atrial fibrillation prevalence according to DM duration in the NOMED-AF population.

The mean duration of diabetes mellitus among the analysed population was 12.0 ± 0.35 years. In 5-year age groups, we found no significant impact on AF prevalence in relation to diabetes duration, regardless of the arrhythmia type (symptomatic AF, SAF, AF de novo). AF prevalence according to DM time course is shown in Fig. 1.

### DM treatment regimen

There were no differences in the course of DM treatment among the study population with and without concomitant AF (oral medication vs insulin vs both oral + insulin): 72% (95% CI: 68.7–75.1) received only oral hypoglycaemic medication; 12.9% (95% CI: 10.7–15.5) was taking both oral treatment + insulin injections; and 15.1% (95% CI: 12.7–17.8) were administered with insulin only.

### Metabolic control

**Figure 2:**
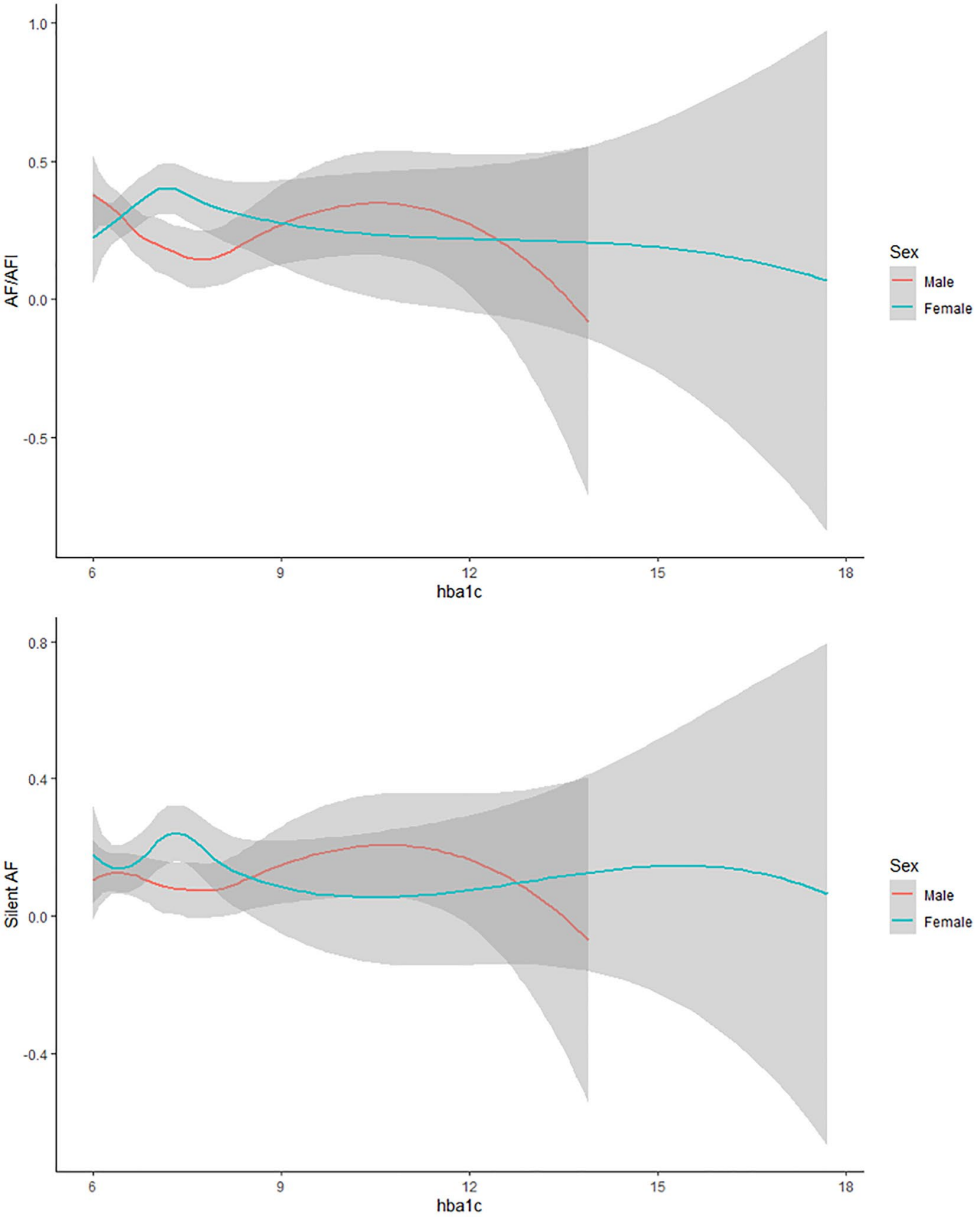
Atrial fibrillation and silent atrial fibrillation prevalence according to HbA1c serum level.

Considering treatment patterns, patients on insulin injections were associated with a higher average level of HbA1c compared to those on oral medication. Metabolic control reflected by HbA1c levels showed no significant association with AF and SAF prevalence, as shown in Fig. 2.

### Multivariate analysis

On multivariate analyses, age (5-year age interval groups)(Odds Ratio (OR) 1.35, 95%CI: 1.18–1.53, p < 0.001), p = 0.042), body mass index (BMI; OR 1.043, 95%CI: 1.01–1.08, p = 0.027) and LDL < 100 mg/dl (OR 0.64, 95%CI: 0.42–0.97, p = 0.037) were independent risk factors for AF prevalence overall (Table 2), while age (OR 1.45, 95%CI: 1.20–1.75, p < 0.001), LDL < 100 mg/dl (OR 0.43, 95%CI 0.23–0.82, p = 0.011), use of statins (OR 0.51, 95%CI: 0.28–0.94, p = 0.031) and HbA1c ≤ 6.5 (OR 0.46, 95%CI: 0.25–0.85, p = 0.013) were associated with silent AF prevalence (Table 2).

**Table 2:** Multivariate analysis of AF and SAF risk factors among people with concomitant DM in the NOMED-AF population.

Risk factors	AF risk in the Polish population with concomitant DM		
	OR	95%CI	p
Sex	1.215	0.846–1.745	0.292
Age^a^	1.345	1.181–1.533	** < 0.001**
Treatment regimen—insulin only	1.248	0.74–2.104	0.407
Treatment regimen—oral medication + insulin	0.584	0.293–1.164	0.127
DM duration^a^	0.986	0.894–1.087	0.774
LDL < 100 mg/dl	0.637	0.417–0.972	**0.034**
Hypertension	0.959	0.443–2.073	0.915
BMI	1.043	1.005–1.083	**0.027**
HbA1c < 6.5	0.836	0.552– 1.265	0.396
HbA1c ≥ 7.5	0.771	0.467 –1.273	0.309
ACEI/ARB	1.259	0.810–1.958	0.306
Statins	0.755	0.496–1.149	0.190

Risk factors	SAF risk in the Polish population with concomitant DM		
	OR	95%CI	p
Sex	1.630	0.940–2.827	0.082
Age^a^	1.448	1.196–1.752	** < 0.001**
Treatment regimen—insulin only	1.446	0.714–2.929	0.306
Treatment regimen—oral medication + insulin	0.565	0.193–1.655	0.297
DM duration^a^	0.949	0.818–1.101	0.491
LDL < 100 mg/dl	0.432	0.227–0.823	**0.011**
Hypertension	1.012	0.346–2.961	0.982
BMI	1.053	0.995–1.115	0.074
HbA1c < 6.5	0.459	0.248–0.850	**0.013**
HbA1c ≥ 7.5	0.618	0.304–1.253	0.182
ACEI/ARB	0.832	0.441–1.568	0.569
Statins	0.513	0.279–0.942	**0.031**

Significant values are in [bold].

ACEI, angiotensin-converting enzyme; ARB, angiotensin receptor blocker; BMI, body mass index; LDL, low-density lipoprotein.

^a^5-year age group.

## Discussion

Our major findings in this prospective, cross-sectional, observational study in the Polish population with concomitant DM are as follows: (1) we found no association between either duration of diabetes mellitus or various antihyperglycemic treatment regimens or glycaemic control on AF and SAF prevalence; and (2) independent predictors of AF were age, BMI and low LDL levels where SAF risk factors were the use of statins and HbA1c ≤ 6.5).

Prior studies have been recently published on the impact of diabetes on AF prevalence^23,24^. Nonetheless, none of the previous research was based on epidemiological population-based screening research, including a representative nationwide sample and using long-term non-invasive ECG monitoring to detect arrhythmia (including silent AF).

We previously reported on increased AF prevalence among individuals with concomitant DM compared to the general Polish population^22^. In the current study, we did not find a significant association between DM duration and AF prevalence in both males and females. Conversely, in a population-based case–control study of newly diagnosed AF, Dublin et al.^8^ described increased AF risk along with the duration of treated diabetes (OR 1.03; 95%CI 1.01–1.06). In a study conducted on the Korean population using National Health Insurance data, there was an increased risk of arrhythmia across the time course of type 2 diabetes^25^. One large Spanish study indicated no apparent impact of diabetes duration or various drug therapy on AF incidence^26^. We also found no differences in AF prevalence between those on oral medication and those on insulin injections or combined therapy, including both methods. Nonetheless, Alves-Cabratosa et al.^26^ showed that particular methods of diabetes treatment did not reach significance in terms of new-onset AF incidence.

It has been speculated that metabolic control, as reflected by HbA1c levels might be associated with an increased risk of AF in subjects with diabetes. Our study demonstrated no significant association—neither lack of metabolic control (HbA1c ≥ 7.5) nor strict glycaemic control (HbA1c ≤ 6.5) on AF prevalence. However, on multivariate analysis, we found that tight glycaemic control expressed by HbA1c ≤ 6.5 was associated with reduced SAF prevalence. Some studies have suggested a relationship between elevated HbA1c levels and AF development^8,27^, while other analyses found no influence^16,26,28^ implying the possible role of glycaemic fluctuations rather than stable HbA1c serum level.

Multivariate regression analyses point out that modifiable risk factors such as raised BMI or proper LDL serum level < 100 mg/dl may contribute to the risk of prevalent AF. These outcomes are in accordance with several studies showing how overweight and obesity significantly increase the risk of AF prevalence^29,30^. There is also epidemiological data showing an inverse relationship between LDL levels and prevalent AF, and our data are consistent with this association. The importance of lipid metabolism in arrhythmia prevalence was also shown by the fact that the use of statins was significantly associated with reduced risk of asymptomatic AF. Nonetheless, for the first time, we report the association with SAF given our study novelty of continuous AF monitoring with a vest-based system.

Given the findings of our study, diabetes mellitus per se might not significantly impact AF prevalence in patients aged ≥ 65 with concomitant DM. The development and progression of this arrhythmia appear to be multifactorial and represented by a cluster of disorders contributing to metabolic syndrome^31,32^. Indeed, several studies have shown that intensive glycaemic control did not independently affect cardiovascular risk reduction and overall mortality^15,33,34^. Hence, we need to strive for weight loss, blood pressure reduction, and dyslipidemia treatment along with glucose-lowering therapies to slow down the progress of vascular complications in these individuals^35,36^. Although intensive glucose lowering decreases the risk of microangiopathy, it does not affect the likelihood of macroangiopathy development^37^. The issue of AF risk reduction in patients with concomitant DM certainly requires further studies, but it seems that a holistic approach comprising comprehensive risk factors management would play a pivotal role^38^. Such attitude has been emphasized as part of the broad characterization and evaluation^39^, followed by a holistic or integrated care approach to AF management^40^, which is now recommended in guidelines^41,42^ given the improved outcomes by adherence with such an approach^43^.

### Strengths and limitations

To the authors best knowledge, this is the first nationwide observational epidemiological study analysing the influence of diabetes duration, therapy and metabolic control on AF prevalence with the use of long-term ECG monitoring. Moreover, study participants were enrolled at random manner from the general population who constitute a representative sample contributing to objectivity and reducing possible bias. In addition, the authors evaluated the influence of disease duration, treatment pattern and its effectiveness, which is novel and relevant in the holistic management of subjects with concomitant diabetes in everyday clinical practice.

Nevertheless, we are aware of some limitations. Although the participants were randomly selected, the response rate was moderate, which could impact a selection bias. Due to the fact that apparently healthier patients are more prone not to respond, the study response rates may be underestimated rather than overestimated in AF prevalence. Furthermore, the limited number of participants in the elderly groups of patients with concomitant DM and AF may also influence a possible bias. What is more, all participants had blood tests taken, including HbA1c levels and fasting plasma glucose. Thus, it is less likely that the population might have included a few patients with DM who remain undiagnosed. The analyses were based on a single HbA1c measurement. Therefore, we were not able to evaluate the impact of long-term glycaemic fluctuations. Moreover, due to lack of sufficient data the authors were not able to analyse the impact of various oral antihyperglycemic drugs on the AF and SAF prevalence. As a result of the study design it could be extraordinarily challenging to perform detailed echocardiography among such a large study population and therefore the authors decided not to include those data in the study protocol. Eventually, the study is based on a national representative sample from the Polish population. Consequently, the results refer to the Polish population and can be directly applied to Polish inhabitants only, who are ethnically homogenous and mainly Caucasians with universal access to the healthcare^18^.

## Conclusions

Diabetes duration, diabetic treatment pattern, or metabolic control per se did not significantly impact the prevalence of AF, including silent AF detected by prospective continuous monitoring. Independent predictors of AF were age, BMI, and low LDL levels, with statins and HbA1c ≤ 6.5 being additional independent predictors for silent AF.

### Supplementary Information

Supplementary Information.

## Data Availability

The datasets used and analysed during the current study are available from the corresponding author on reasonable request.

## Abbreviations

AF: Atrial fibrillation
BMI: Body mass index
CABG: Coronary artery bypass graft surgery
CHA_2_DS_2_-VASc: Clinical prediction score for stroke risk evaluation in patients with atrial fibrillation (congestive heart failure, hypertension, age ≥ 75, diabetes mellitus, prior stroke or thromboembolism, vascular disease, age 65–74, sex category)
DM: Diabetes mellitus
ECG: Electrocardiography
HbA1c: Haemoglobin A1c
PAD: Peripheral arterial disease
PCI: Percutaneous coronary intervention
SAF: Silent atrial fibrillation
SD: Standard deviation
95% CI: 95% confidence interval
