# Supplementary material for: Impact of diabetes mellitus severity, treatment regimen and glycaemic control on atrial fibrillation prevalence in the Polish population aged ≥ 65
Source: Sci Rep. 2023 Oct 12;13:17252. doi: 10.1038/s41598-023-43939-5 (PMC10570325; doi:10.1038/s41598-023-43939-5)
Supplement: Supplementary file 1 — Supplementary Information. [file 41598_2023_43939_MOESM1_ESM.docx]

**Supplementary Appendix**

**Impact of diabetes mellitus severity, treatment regimen and glycaemic control on atrial fibrillation prevalence.**

A report from the Non-invasive Monitoring for Early Detection of Atrial Fibrillation (NOMED-AF) prospective cross-sectional observational study

Jakub Gumprecht ^a, c^, Gregory Y.H Lip ^a, b^, Adam Sokal ^d^, Beata Średniawa ^c,d,j^, Jakub Stokwiszewski ^e^, Marcin Rutkowski ^i^ , Tomasz Zdrojewski ^i^ , Tomasz Grodzicki ^f^ , Jarosław Kaźmierczak ^g^ , Grzegorz Opolski ^h^, Zbigniew Kalarus ^c, d, j^

^a^ Liverpool Centre for Cardiovascular Science, University of Liverpool and Liverpool Heart & Chest Hospital, Liverpool, United Kingdom, ^b^ Aalborg Thrombosis Research Unit, Department of Clinical Medicine, Aalborg University, Aalborg, Denmark, ^c,^ DMS in Zabrze, Medical University of Silesia, Department of Cardiology, ^d^ Department of Cardiology, Silesian Centre for Heart Diseases, Zabrze, Poland, ^e^ National Institute of Hygiene, Poland, ^f^ Department of Internal Medicine and Gerontology, Jagiellonian University Medical College, Kraków, Poland ^g^ Department of Cardiology, Pomeranian Medical University, Szczecin, Poland, ^h^ First Chair and Department of Cardiology, Medical University of Warsaw, Poland, ^i^ Department of Preventive Medicine and Education Medical University of Gdansk, ^j^ Silesian Park of Medical Technology Kardio-Med Silesia in Zabrze, Poland

# **Study sample selection procedure**

The sample consisted of 3,014 randomly chosen individuals, representative of the Polish general, noninstitutionalized population aged 65+. Multistage, stratified and clustered sampling procedure was used.

During the first stage of the sampling procedure, 59 strata were created by dividing each of 16 provinces (voivodships) into up to four categories of municipalities: villages, towns with population: <50,000, 50,001-200,000 and over 200,000. The final number of strata was lower than theoretically possible 64 because not all provinces municipalities of the highest population category existed. The number of respondents to draw in each stratum was set proportional to the size of its population aged 65+

During the second stage of sampling, individual municipalities were drawn in each of the previously defined strata separately, with the probability proportional to the municipality's population size. In total, 137 municipalities were selected from the complete list of 3 118 Polish municipalities. Finally, each municipality was divided into territorial clusters of approximately similar population size. Then the clusters were randomly selected in each municipality. The clustering was aimed to lower the cost of the study.

The third stage of the sampling procedure was selecting individual respondents within previously drawn clusters. The sampling frame consisted of all individuals living in selected clusters aged 65+. recorded in the PESEL database (national registry covering all Polish citizens). In each of the age categories (65-69, 70-74, 75-79, 80-84, 85-89 and 90+ years) similar numbers of men and women were selected. This resulted in oversampling of older age groups. This was done to ensure that the size of the final subsample of the eldest subjects will be enough for separate analyses. The oversampling was corrected at the stage of statistical analysis with weights to get population estimates.

For each of the 3000 participants, another 9 subjects living in the same cluster were drawn. These “spare” addresses were used only if the address of the primarily chosen subject was incorrect or an individual refused to take part in the study.

Inability to answer the questionnaire by patients (i.e., because of dementia) did not exclude them from the study. In such cases, caregivers or close family members were asked to provide information. This was done to avoid selection bias towards the healthier part of the population. Patients with already diagnosed AF were included. The only exclusion criterium was lack of patient’s consent or infrequent situation of local environment potentially dangerous for the study nurse.

There were attempts to contact 10 425 respondents. Among them, 7 429 cases were eligible to participate. The remaining 2 996 cases were confirmed invalid or outdated addresses (542), subject deceased before contact attempt (357), was away during the study period (281), or nurse was unable to contact patient despite three attempts (1 816). The final number of the interviewed patients was 3 014, resulting in a response rate of about 41%. The group of non-respondents consisted of subjects who refused to take part in the study (3 602), whose family refused (742), or did not participate for other reasons (71). Long term ECG monitoring was performed in the final sample of adults aged ≥65 years (n = 3 014) (eFigure 1).

**eFigure 1. NOMED-AF – a flow of the study**

4415 non-participants:

- 3602 – refusal of participant
- 742 – refusal of participant’s family member
- 71 – other reasons

2996 non eligible:

- 303 – correct address, resident has moved out prior to the study
- 1816 – ‘closed door’ (any contact with the resident impossible during study period)
- 74 – incorrect address
- 165 – resident dwelling in a hospital,

a nursing home or a health care centre

- 281 – resident away, not present during study period
- 357 – died before start of the to survey
